# Estonian Dental Students’ Oral Health-Related Knowledge, Attitudes and Behaviours (KAB): National Survey-Based Study

**DOI:** 10.3390/ijerph19031908

**Published:** 2022-02-08

**Authors:** Abanoub Riad, Ave Põld, Jana Olak, Hans-Peter Howaldt, Miloslav Klugar, Martin Krsek, Sameh Attia

**Affiliations:** 1 Department of Public Health, Faculty of Medicine, Masaryk University, Kamenice 5, 62500 Brno, Czech Republic; klugar@med.muni.cz (M.K.); krsek@med.muni.cz (M.K.); 2 Czech National Centre for Evidence-Based Healthcare and Knowledge Translation (Cochrane Czech Republic, Czech EBHC: JBI Centre of Excellence, Masaryk University GRADE Centre), Faculty of Medicine, Institute of Biostatistics and Analyses, Masaryk University, 625 00 Brno, Czech Republic; 3 Department of Family Medicine and Public Health, Faculty of Medicine, University of Tartu, Ravila 19, 50411 Tartu, Estonia; avepold@gmail.com; 4 Institute of Dentistry, Faculty of Medicine, University of Tartu, Ravila 19, 50411 Tartu, Estonia; jana.olak@ut.ee; 5 Department of Oral and Maxillofacial Surgery, Justus-Liebig-University, Klinikstrasse 33, 35392 Giessen, Germany; hp.howaldt@uniklinikum-giessen.de

**Keywords:** dental education, dental students, Estonia, health knowledge, attitudes, practice, Hiroshima University Dental Behavioural Inventory, HU-DBI, oral health, oral hygiene

## Abstract

The strategic plan for dentistry and oral health in Estonia of 2030 focuses on oral health promotion and disease prevention through undergraduate dental curricula and fostering public health-oriented research among students. The present study was designed as a descriptive cross-sectional study to evaluate oral health-related knowledge, attitudes, and behaviours (KAB) of dental students in Estonia. The study was carried out in the spring semester of 2020, and it used a modified version of the Hiroshima University Dental Behavioural Inventory (HU-DBI). A total of 129 students responded to the survey, constituting a response rate of 93.5% due to the total population sampling (census) technique used in this study and the small target population size. Out of the 124 students included in the final analysis, 79% were females, 62.1% were clinical students, 11.3% reported smoking tobacco at least once a week, and 86.3% reported problematic internet use. The present study found that mean HU-DBI score of Estonian dental students was 8.09 ± 1.22 which is so far the highest recorded HU-DBI score in Europe. There was no significant difference between female vs. male or preclinical vs. clinical students in terms of HU-DBI score. While clinical students reported less faulty oral hygiene practices, such as hard toothbrush use and aggressive toothbrushing, preclinical students reported a slightly higher mean HU-DBI score. Smoking behaviour was more common among male and clinical students, and it was also associated with alcohol drinking and worry about teeth colour and halitosis.

## 1. Introduction

The strategic plan for dentistry and oral health in Estonia for 2030 was recently completed by the Estonian Dental Association [1]. Compared to the previous 10-year plan for 2020, this document emphasises even further the connections between oral health and general health and puts disease prevention to the centre of Interprofessional collaboration and dental education [2]. It echoes global targets set by the Council of European Dentists (CED) and the World Dental Federation (FDI) to ensure the accessibility to needs-based and people-centred quality oral health care [3].

Comparing the 10-year vision plans, a clear shift can be seen from a mainly treatment-oriented role of dentists towards an oral health promoter role by 2030. Objectives include promoting optimal oral hygiene practices and the use of fluoride toothpaste, offering diet and smoking cessation counselling [1,3]. The strategic plan urges to focus more on disease prevention in the undergraduate dental curriculum and foster public health-oriented research among students [1]. More upstream interventions such as sugar taxation are also advised looking into the future. A very important goal is lowering the caries index of children in all age groups to continue the progress towards an ultimate goal- a generation of children with healthy teeth [1].

Clearly enough, the structure of the dental curriculum is an essential element influencing the establishment of a dental workforce that enables achieving the targets set for 2030. The current dental curriculum of the University of Tartu includes disease prevention and oral health promotion courses starting from the first year and extending into the advanced years of studies [4,5,6,7]. The preclinical years (first- and second-year) have a greater emphasis on prevention and promotion, e.g., Promotion of Oral Health (MVST.00.006) and Health Promotion (ARTH.04.044), while related subjects continue during the clinical study years (third-, fourth-, and fifth-year), e.g., Prevention of Oral and Dental Diseases (ARST.01.100) [4,5,6,7]. Courses about oral health can influence and improve the personal oral health attitudes and behaviours of dental students and support knowledge sharing with patients [8,9,10].

In 1988, Makoto Kawamura developed a psychometric instrument that evaluates oral health-related knowledge, attitudes and behaviours (KAB) among dental students, which is referred to as Hiroshima University Dental Behavioural Inventory (HU-DBI) [9,10]. Since its introduction, the HU-DBI has undergone multiple testing processes for its internal and external validity and exhibited excellent psychometric properties [10,11,12]. Therefore, it has been used in hundreds of studies targeting dental students in several countries, including European ones, e.g., Switzerland, the Netherlands, Portugal, the United Kingdom, Poland, and Finland [13,14,15,16,17].

The overarching aim of this study was to evaluate oral health-related KAB of Estonian dental students. The primary objective was to assess the levels of oral health KAB using HU-DBI, while the secondary objectives were: i) to explore the sociodemographic and behavioural determinants of oral health KAB among Estonian dental students, and ii) to explore the predictors of smoking behaviour among Estonian dental students.

## 2. Materials and Methods

### 2.1. Design

A descriptive cross-sectional survey-based study was carried out during the spring semester of 2019/2020 among dental students of the University of Tartu (Tartu, Estonia). The study was designed and reported following the STrengthening the Reporting of OBservational studies in Epidemiology (STROBE) guidelines for cross-sectional studies [18]. (Supplementary Materials).

### 2.2. Participants

In Estonia, only one dental school awards doctor of dentistry “DD” degrees nationwide [19]. The sole Estonian school is located at the University of Tartu, and it had a total of 138 students enrolled during the academic year 2019/2020 [20]. Given the small target population size, the present study followed a “census” technique or a “total population sampling” method because all dental students in Estonia were approachable [21]. Brief presentations about the study objectives, methodology, and participation benefits and risks were held after each academic level’s lectures, i.e., first-, second-, third-, fourth- and fifth-year. The students were offered to share all their potential inquiries to clarify any doubts about the study context or objectives prior to participation.

**Figure 1:**
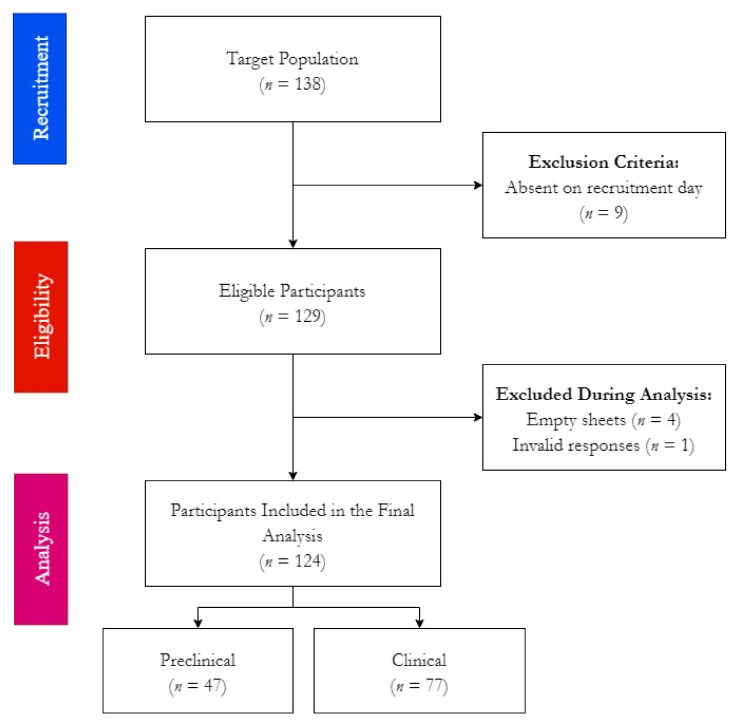
Workflow of Estonian dental students’ survey for oral health knowledge, attitudes, and behaviours, Spring 2020.

Following the introductory presentations, the survey was distributed in paper form among the target participants, and they were offered to withdraw from the study at any point before handing back their final answers. While nine students were absent when the introductory presentations were held, all attending students handed back their survey sheets. A total of 129 sheets were received, representing a response rate of 93.5%, whilst five of these 129 sheets were empty or invalid due to missing sociodemographic data, i.e., gender or academic level, thus leading to a completion rate 96.1%. (Figure 1)

### 2.3. Instrument

The present study used a self-administered questionnaire (SAQ) to collect data from the target participants. The SAQ comprised three basic categories, i) sociodemographic characteristics, which included gender and academic level, ii) original items of HU-DBI, which are twenty dichotomous items (agree/disagree) inquiring about oral health KAB, and iii) general health behaviours that included tobacco smoking “I consume tobacco at least once a week”, alcohol drinking “I drink alcohol at least once a week”, problematic internet use “I find myself using my smartphone/computer longer than I planned”, and regular dental check-up “I go to the dentist/hygienist for a regular check-up at least once a year” [22]. (Appendix A).

The scoring system of HU-DBI implies that one point is given for each agreement answer to questions no. 4, 9, 11, 12, 16, and 19 and each disagreement answer of the questions no. 2, 6, 8, 10, 14, and 15. The overall score of HU-DBI ranges between 0 (worst KAP score) and 12 (best KAP score). The sum of items no. 2, 8, 10, 15, and 19 represents the score of the oral health knowledge domain, while the sum of items no. 6, 11, and 14 is the oral health attitudes score, and the sum of items no. 4, 9, 12, and 16 is the oral health behaviours score [23].

The English version of HU-DBI had been used in this study, as no validated Estonian version was available when the study was conducted. According to the English Proficiency Index (EPI) of Education First (EF) of 2021, Estonia is ranked as a “high proficiency country”, which implies that Estonian adults’ average score of the Common European Framework of Reference for Languages (CEFR) is B2 [24]. Therefore, there was no significant need to produce an Estonian version of HU-DBI while the target population is a highly educated subset, i.e., dental students.

### 2.4. Ethics

The study protocol was fully reviewed and approved on 20 November 2019 by the Ethics Committee of the Faculty of Medicine, Masaryk University, under reference no. 48/2019. All participants had to provide their informed consent before responding to the SAQ. The present study had been carried out in compliance with the declaration of Helsinki for research involving human subjects and the European Union (EU) General Data Protection Regulation (GDPR) for storing and managing data [25,26].

The study did not collect identifying personal data; therefore, retrospective identification of the participants was not possible because the amount of collected personal data was inadequate. The participants did not receive incentives to participate in the study, and no penalties were imposed for the non-participants.

### 2.5. Analyses

The Statistical Package for the Social Sciences (SPSS) version 28.0 (SPSS Inc. Chicago, IL, USA, 2021) was used in all statistical tests [27]. Categorical variables such as gender, academic level, clinical experience and dichotomous items were summarized using frequencies (n) and percentages (%). Numerical variables such as HU-DBI score and its domains score, e.g., knowledge score, attitudes score, and behaviours score, were summarized with mean and standard deviation (µ ± SD). Given the small sample size, inferential tests such as Fisher’s exact test, independent-samples proportions test, two-sample *t*-test, Chi-squared test (χ2), and Jonckheere-Terpstra test (JT) were used with a significance level of < 0.05. A binary logistic regression was performed to verify the various sociodemographic and behavioural predictors of tobacco smoking.

## 3. Results

### 3.1. Demographic Characteristics

A total of 124 students were included in this study, out of which 79% were females and 21% were males. The recruited sample tended to be equally distributed over the five years, with fifth-year being the most represented year (24.2%) and first-year is the least represented (16.9%). Clinical students (3rd, 4th, and 5th level) represented 62.1% of the recruited sample, while the rest were preclinical students (1st and 2nd level) (Table 1).

### 3.2. Health Behaviours

Overall, fourteen students (11.3%) reported smoking tobacco at least once a week, twenty students (16.1%) reported drinking alcohol at least weekly, and 107 students (86.3%) reported problematic internet use. Male students had a significantly higher level of tobacco smoking (*α*. < 0.001) than their female peers, 34.6% vs. 5.1%, respectively. Similarly, male students had a significantly higher level of alcohol drinking (*α*. = 0.034) than females, 30.8% vs. 12.2%, respectively. Clinical students had higher levels of tobacco smoking (15.6% vs. 4.3%) and alcohol drinking (20.8% vs. 8.5%) than preclinical students. Problematic internet use was not significantly different between females (86.7%) and males (84.6%); however, it was significantly more reported by clinical students (92.2%) than preclinical students (76.6%). The regular dental check-up habit was not different among females and males, whilst it was more common among preclinical (95.7%) than clinical students (88.3%) (Table 2).

### 3.3. HU-DBI Responses

Items no. 2 (bleeding gingiva) and no. 15 (postponing of dental visits) had the lowest agreement level, which was 2.4% for each of them. On the other hand, item no. 9 (careful toothbrushing) had the highest agreement level of 95.2%, followed by item no. 12 (post-brushing checking) 76.6%, item no. 20 (positive dentist’s feedback) 71.8%, and item no. 1 (not worrying about dentist visiting) 68.5%.

The items related to faulty oral hygiene practices, such as item no. 17 (using a toothbrush with hard bristles), and item no. 18 (aggressive toothbrushing), had low agreement levels of 9.7% and 7.3%, respectively. About 83.1% of the participants reported receiving oral hygiene training by a professional (item no. 10), and 54.8% reported using disclosing agents to check dental plaque (item no. 16). Above half of the participants (51.6%) were worried about their teeth colour (item no. 3), and almost two-thirds (66.9%) were worried about having halitosis (item no. 13). Only 14.5% of the participants were not satisfied with their gingival color (item no. 7), whilst 58.1% believed toothbrushing alone could prevent periodontal disease (item no. 14).

The items used to reflect the highest possible levels of oral hygiene awareness, such as item no. 5 (using a child-sized toothbrush) and item no. 11 (brushing without toothpaste), have low agreement levels of 9.7% and 13.7%, respectively (Table 3).

#### 3.3.1. Gender

There was no single significant difference between females and males in terms of any item on comparing responses across genders. Nevertheless, females had a higher agreement level with item no. 5 (using a child-sized toothbrush) than males, 11.2% vs. 3.8%, respectively. Similarly, items no. 11 (brushing without toothpaste) and no. 12 (post-brushing checking) had a high higher agreement among females (16.3% and 79.6%) than their male colleagues (3.8% and 65.4%), respectively (Table 4).

#### 3.3.2. Academic Level

The fifth-year students (seniors) reported a significantly higher agreement level (*α* = 0.031) with item no. 16 (using disclosing agent) than first-year students (freshers), 73.3% vs. 47.6%, respectively. Contrarily, freshers had a significantly higher agreement level (*α*. = 0.032) with item no. 17 (using a toothbrush with hard bristles) than seniors, 19% vs. 3.3%, respectively. Similarly, item no. 18 (aggressive toothbrushing) had a significantly higher agreement (*α*. = 0.032) among freshers (19%) than seniors (3.3%). Moreover, item no. 14 (preventing periodontal disease by brushing alone) had a significantly higher agreement level (*α*. < 0.001) among seniors than freshers, 56.7% vs. 9.5%, respectively. The rest items did not have significant differences between freshers and seniors; nevertheless, inter-year differences were significant for a few items, e.g., item no. 1 (first- vs. fourth- year), item no. 5 (second- vs. third- year), item no. 13 (first- vs. second-year), and item no. 20 (second- vs. fourth-year) (Table 4).

#### 3.3.3. Clinical Experience

Clinical students had a significantly higher agreement level (*α*. = 0.032) with item no. 14 (preventing periodontal disease with toothbrushing alone) than preclinical students, 49.4% vs. 29.8%, respectively. Similarly, clinical students had a significantly higher agreement level (*α*. = 0.032) with item no. 20 (positive dentist’s feedback) compared with their preclinical colleagues, 77.9% vs. 61.7%, respectively. Preclinical students had a higher agreement with hazardous items such as no. 17 (using a toothbrush with hard bristles) 71% vs. 5.2%, and no. 18 (aggressive toothbrushing) 12.8% vs. 3.9%, respectively, without statistical significance. Notably, preclinical students had a higher agreement with item no. 5 (using a child-sized toothbrush) than clinical students, 17% vs. 5.2%, respectively.

### 3.4. HU-DBI Scores

The mean overall HU-DBI score was 8.09 ± 1.22 (Range: 5–11), with a mean knowledge score of 4.07 ± 0.65 (R: 2–5), attitudes score of 1.55 ± 0.68 (R: 0–3), and behaviours score of 2.47 ± 0.76 (R: 1–4). Female students had an insignificantly higher HU-DBI score (8.16 ± 1.23) than their male colleagues (7.81 ± 1.13). The gender-based differences were more expressed in the oral health attitudes and behaviours domains than the knowledge domain.

**Figure 2:**
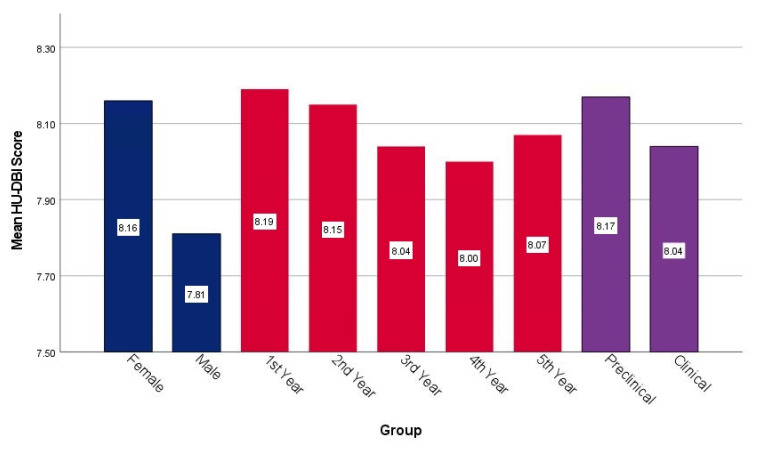
Mean HU-DBI score of Estonian dental students stratified by gender, academic level and clinical experience, Spring 2020 (*n* = 124).

The overall HU-DBI score decreased steadily from first-year (8.19 ± 1.47) to fourth-year (8.00 ± 1.09), then it increased again in the fifth-year (8.07 ± 1.05). Freshers had a slightly higher attitudes score (1.71 ± 0.64) than seniors (1.47 ± 0.63), whilst seniors had a slightly higher behaviours score (2.60 ± 0.50) than freshers (2.38 ± 0.81) (Figure 2).

**Figure 3:**
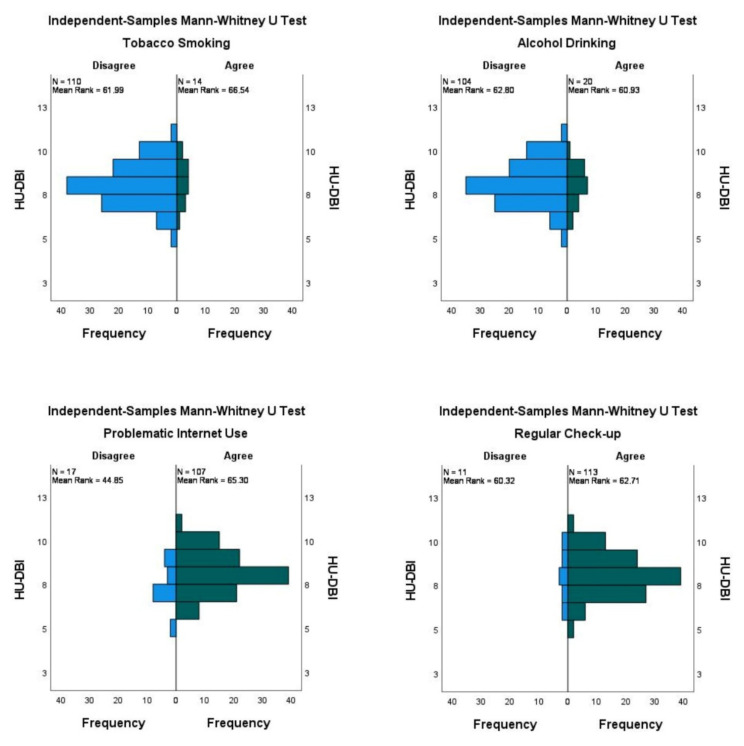
Mean rank of HU-DBI score of Estonian dental students stratified by tobacco smoking, alcohol drinking, problematic internet use, and regular dental check-up, Spring 2020 (*n* = 124).

Preclinical students had higher HU-DBI scores (8.17 ± 1.36) than their clinical colleagues (8.04 ± 1.13) without statistical significance. While knowledge and attitudes scores were higher among preclinical students, behaviours score was higher among clinical students. Interestingly, the students who reported smoking tobacco at least once a week had a slightly higher HU-DBI score (8.21 ± 1.19) than those who were nonsmokers (8.07 ± 1.22). Contrarily, the students who reported drinking alcohol at least once a week had a slightly lower HU-DBI score (8.00 ± 1.08) than non-drinkers (8.11 ± 1.25) (Figure 3).

The participants with reported problematic internet use had a significantly higher HU-DBI score (*α*. = 0.006) and knowledge score (*α*. = 0.008) than their counterparts. The students who reported undertaking regular dental check-ups had a slightly higher HU-DBI score (8.10 ± 1.20) (Table 5).

### 3.5. Year-Over-Year Analysis

The year-over-year (YOY) analysis aimed to evaluate the inter-year differences using pairwise comparison (Mann–Whitney *U* test). The YOY analysis revealed no significant difference in oral health knowledge score, attitudes score, behaviours score, or overall HU-DBI score among any of the paired years (Table 6).

### 3.6. Tobacco Smoking

On comparing individual items responses between smokers and nonsmokers, a few items were significantly different, and several others showed a trend favouring nonsmokers. The items no. 3 (worrying about teeth colour) and no. 13 (worrying about halitosis) were significantly (*α*. = 0.007 and 0.034) more common among smokers (85.7% and 92.9%) than nonsmokers (47.3% and 63.6%), respectively. Nonsmokers had lower levels of dental anxiety (item no. 1), gingival bleeding (item no. 2), and using toothbrushes with hard bristles (item no. 17) than smoker participants without a statistical significance (Table 5).

Logistic regression analysis for the predictors of tobacco smoking revealed that male gender (AOR: 8.84), clinical students (AOR: 2.73), alcohol drinking (AOR: 3.32), and problematic internet use (AOR: 1.58) had higher odds for smoking behaviour. Given the results of the univariate analysis, i.e., chi-squared and Fisher’s exact tests, the items no. 3 (worrying about teeth colour) and no. 13 (worrying about halitosis) were used in the logistic regression model to predict smoking behaviour. Agreement with item no. 3 (AOR: 5.41) and item no. 13 (AOR: 3.15) had higher odds for reporting tobacco smoking (Table 7).

The proposed model managed to predict 90.3% of the participants’ smoking status correctly; and the Nagelkerke’s pseudo R^2^ of the proposed model was 0.426 (Table 8).

## 4. Discussion

The present study found that the mean HU-DBI score of Estonian dental students was 8.09 ± 1.22, which is the highest recorded HU-DBI score in Europe. Prior studies revealed that the HU-DBI score of dental students in Switzerland was the highest (8.02 ± 1.27), followed by the Netherlands (8.0 ± 1.19), Portugal (7.74 ± 1.40), the United Kingdom (7.33), Poland (7.23 ± 1.45), and Finland (7.15 ± 1.13) [13,14,15,16,17]. On the other hand, the lowest HU-DBI score of European dental students was reported in Lithuania (6.35 ± 1.43), Croatia (6.62 ± 1.54), and Greece (6.86 ± 1.83) [28,29,30]. Kawamura et al. 2005 concluded that cultural orientations might yield valuable results in trans-national comparisons of oral health KAB, especially among dental students [11].

Over half of our participants (54.8%) reported using disclosing agents to visualize their dental plaque, which is similar to the findings of dental students in the United Kingdom (55%) and Switzerland (52.1%), whilst it is significantly higher than Serbia (3%), Croatia (23.1%), Poland (29.6%), and Greece (33%) [13,15,16,29,30,31]. The use of plaque-disclosing agents is recommended for enhancing patients’ motivation towards oral hygiene [32]. Recent systematic reviews showed that disclosing agents can help improve plaque control practices of children, adolescents, patients with intellectual disabilities and orthodontic patients [33,34,35]. The prevalence of disclosing agent use of Estonian dental students was higher among seniors (73.3%) as compared with freshers (47.6%), which reflects a significant improvement occurring with progressing in dental education that was found in multiple HU-DBI-based studies, e.g., Croatia (seniors: 42.6% vs. freshers: 16.1%), the United Kingdom (78% vs. 33%), Romania (58% vs. 26%), and Poland (33.9% vs. 1.9%) [15,16,36,37]. Notably, the freshers in Estonia (47.6%) had the highest disclosing agent use compared with the freshers of other European countries; thus, suggesting that Estonian youth might have better oral health.

The toothbrushes with small heads are widely recommended for children; therefore, they are referred to as “child-sized toothbrushes” [38,39]. Nevertheless, adults’ use of child-sized toothbrushes is also recommended due to their superior accessibility for posterior teeth [40,41,42,43]. Moreover, the most updated systemic review on toothpaste types revealed moderate evidence suggesting that toothbrushing with toothpaste had no added value for mechanical removal of dental plaque [44]. Therefore, the use of child-sized toothbrushes (item no. 5) and toothbrushing without toothpaste (item no. 11) had been incorporated in HU-DBI as signals for excellent oral hygiene-related awareness and practice [9,10]. In our sample, only 9.7% and 13.7% reported using child-sized toothbrushes and toothbrushing without toothpaste, respectively. Other European HU-DBI-based studies of dental students showed much lower levels of child-sized toothbrush use, e.g., Switzerland (4.1%), Finland (4%), Poland (1.8%) [13,16,17]. Similarly, toothbrushing without toothpaste was also less common in the United Kingdom (6%) [15].

Dental anxiety is defined as “patient’s response to the stress specific to the dental context”; it is a key barrier for seeking oral healthcare [45]. About 68.5% of our participants reported being not worried about visiting the dentist (item no. 1), which is comparable to the results of dental students in Switzerland (64.7%), Finland (60%), and the United Kingdom (70%) [13,15,17]. Storjord et al. 2014 compared the levels of dental anxiety among Norwegian university students and found that dental students had significantly lower anxiety levels than biology and clinical psychology students [46]. On comparing dental anxiety among dental students and allied healthcare students, several studies found that dental students were less affected by dental anxiety than general medicine, nursing, and midwifery students [47,48]. Preclinical students were more anxious within dental schools than clinical students in Bulgaria, Israel, and Pakistan, thus suggesting that dental education can help reduce dental anxiety [49,50,51]. Farooq et al. 2015 concluded that female dental students had significantly higher dental anxiety scores than their male colleagues; while the same trend was found in our sample, it was not statistically significant [50].

Recent systematic reviews found that soft and extra-soft toothbrushes were safer as they caused soft tissue injuries and hard tissue abrasion to a lesser extent than hard toothbrushes, which could be more efficient in plaque removal [52,53]. The prevalence of hard toothbrushes use had significantly declined from first-year (19%) to fifth year (3.3%) among our participants (item no. 17), which is similar to what was previously reported in Finland (15% vs. 0%), Poland (27.8% vs. 1.7%), the United Kingdom (33% vs. 4%), Romania (37% vs. 11%), and Croatia (34.9% vs. 12.5%) [15,16,17,28,37]. Similarly, aggressive toothbrushing had declined among Estonian dental students from first-year (19%) to fifth-year (3.3%) significantly (item no. 18). It is worthy of mentioning that prevalence of hard toothbrushes use was higher among smokers (21.4%) than nonsmokers (8.2%); however, this difference was not statistically significant (*α.* = 0.136).

Dentistry in Estonia had been a highly female-dominated profession, with approximately 82% of all practising Estonian dentists being female, according to the National Institute for Health Development [54]. Our recruited sample reflected female dominance, as 79% of our participants were females and 21% were males. On comparing the HU-DBI responses between females and males, no significant differences were found, which might be attributed to our sample’s limited proportion of males. On the other hand, the overall HU-DBI score was higher among females than their male colleagues (8.16 vs. 7.81), which is consistent with the findings of previous HU-DBI-based studies that confirmed female superiority, e.g., Finland (7.32 vs. 6.83), Poland (7.36 vs. 6.95), Greece (7.13 vs. 6.48), and Croatia (6.58 vs. 6.17) [16,17,28,29].

Although our study did not find significant gender-based differences in oral health KAB of Estonian dental students, those differences exist within the general population. The Estonian Adult Health Behaviour Survey (ETRTU) of 2020 revealed that men brushed their teeth less often than women when comparing the same age groups of men and women [55]. The differences were especially remarkable in the older age groups, as women aged between 45 and 54 were six times more likely to brush their teeth than men. Men also were less likely to visit dentists than women in Estonia; this pattern was sustained in all age and socioeconomic groups [55].

Clinical students predominantly exhibited better HU-DBI scores than their preclinical colleagues in prior HU-DBI-based studies, e.g., Lithuania, Poland, Croatia, and Romania [16,28,30,37]. The commonly adopted hypothesis for this difference is that dental education increases students’ oral health-related knowledge that may positively impact students’ attitudes and behaviours [23]. In contrast to this notion, we found no significant difference between preclinical and clinical students in average HU-DBI scores in Estonia; however, few items improved gradually throughout the five years of study, e.g., item no. 14, 16, 17 and 18. Interestingly, Estonian preclinical students manifested a slightly higher HU-DBI score than clinical students (8.17 vs. 8.04), which can be attributed to the design of the dentistry curriculum at the University of Tartu [4]. During the first year of DD program, the mandatory course (MVST.00.006) of “Promotion of Oral Health” aims to provide students with knowledge of the methods for prevention of oral diseases, educating children about oral health and nutrition, treatment possibilities and compensated oral health services for various age groups [5]. In addition, the first-year dental students in Tartu have to do the course (ARTH.04.044) of “Health Promotion” which is focused on equipping them with the basic knowledge of health promotion principles, Estonian national health plan, leading health and risk behaviours, health promotion interventions [6]. These preventive courses are administered during the first-year alongside the courses of epidemiology, biostatistics, and environmental and occupational health [4]. Preventive education continues throughout the clinical training of Estonian students through the courses of periodontology and paediatric dentistry [4].

Dentists, like other healthcare professionals, have an essential role in primary prevention and health promotion by recommending their patients to adopt healthier lifestyles, which is not limited to oral hygiene practices, but also include smoking cessation, moderate drinking, healthy diet, physical activity, and immunization [56,57,58,59,60,61,62,63,64]. Tobacco use is associated with multiple oral conditions, e.g., periodontal disease, oral cancers, orofacial defects [65]. Estonia had implemented multiple legislative reformations during the last twenty years to cut down the prevalence of tobacco smoking nationwide [66]. According to the Global Health Observatory (GHO), the prevalence of tobacco use among the Estonian population had declined from 36.4% in 2007 to 30.5% in 2018 [67]. Nevertheless, the Estonian Adult Health Behaviour Survey (ETRTU) of 2020 showed that patients across all age groups reported receiving counselling on smoking cessation from their physicians rather than their dentists; thus underlining the need for a more proactive role of Estonian dentists in the fight against tobacco nationwide [55].

In our sample, tobacco smoking was reported by 11.3% of the participants which was significantly lower than what Rodakowska et al. 2020 found among Italian (42%) and Polish (28%) dental students; in spite of the fact that the prevalence of tobacco use among Estonian general population (30.5%) is higher than both Polish (26%) and Italian (23.4%) general populations [67,68,69,70]. The difference between our findings and the findings of Rodakowska et al. 2020 can be attributed to the frequency of smoking that was used to determine smoking behaviour; as in our study we used “once a week” while Rodakowska et al. 2020 used “once during the past 30 days” [68]. Our male students (34.6%) were more inclined to smoke tobacco than females (5.1%), which is in agreement with the Estonian physicians’ smoking levels (men: 14.3% vs. women: 5.2%) [71]. Interestingly, the clinical students were more inclined to be smokers (15.6%) than their preclinical peers (4.3%). These findings are consistent with what was found in Lithuania, Latvia, Germany, and Turkey, as clinical students were more likely to be smokers than preclinical students [30,72,73,74]. The smoking students had higher significantly levels of worry about their teeth colour (item no. 3) and halitosis (item no. 13), which might reflect their increased knowledge about oral consequences of smoking. The study-related stress can help explain this paradox, as tobacco smoking was frequently cited as one of the stress relievers for dental students [75,76]. Logistic regression analysis for the predictors of tobacco smoking also revealed that in addition to being a male clinical student, the odds for being a smoker were higher if a dental student also consumed alcohol and reported to use their smartphone/computer longer than what was initially planned.

An interesting sighting was that problematic internet use was significantly more common among clinical students (92.2%) than preclinical students (76.6%). The students who reported problematic internet use had higher levels of knowledge score, attitudes score, behaviours score and overall HU-DBI score. This finding should be interpreted cautiously because the dimension of problematic internet use that we have used in this study was poor time management “I find myself using my smartphone/computer longer than I planned”, which does not reflect the platforms or activities the students are engaged with while being online [77,78,79]. According to the Internet World Stats (IWS) database, the internet usage penetration in Estonia was 97.9% which makes it the second in the European Union (EU) after Iceland (99%) [80]. A multinational study for European adolescents found that problematic internet use was more common in Estonia than any other European country [81].

### 4.1. Strengths

To the best of the authors’ knowledge, this study is the first to assess oral health KAP of Estonian dental students. Given the high response rate that exceeded 93%, the present study findings’ external validity (generalizability) is substantially high as we almost managed to approach the target population entirely. The study aimed to draw attention to sociodemographic determinants and behavioural correlates of oral health KAP among Estonian dental students.

### 4.2. Limitations

Several limitations should be carefully considered while interpreting the present study’s findings. Firstly, the cross-sectional design is inheritably limited in tracking the changes in the investigated phenomena; therefore, our year-over-year analysis findings should be interpreted cautiously. Secondly, minimal personal information was collected from the participants that did not allow evaluation of the impact of social determinants of oral health such as socioeconomic level and ethnicity on participants’ oral health KAP. Thirdly, the general health behaviours were not evaluated sufficiently because of the need to keep the SAQ as short as possible to secure a high response rate, while noticing that the target population was already small. Fourthly, the study was conducted a couple of years before the time this manuscript was submitted; nevertheless, Estonia’s dental curriculum had not been changed since the data was collected in the spring semester of 2020.

### 4.3. Implications

The findings of this study suggest the need for evaluating oral health KAP of highly educated subsets of the Estonian population, i.e., university students, with a focus on healthcare students. The role of preventive elements in the Estonian dental curriculum should be investigated in-depth because it might serve as a guiding template for other European curricula. Estonian dental curriculum can benefit from integrating smoking cessation within their public health and preventive courses at both theoretical and practical levels. The prevalence of problematic internet use and its impact on adolescents’ health should be further investigated in Estonia, perceived as “Silicon Valley of Europe”.

## 5. Conclusions

The present study found that mean HU-DBI score of Estonian dental students was 8.09 ± 1.22 which is so far the highest recorded HU-DBI score in Europe. There was no significant difference between female vs. male or preclinical vs. clinical students in terms of HU-DBI score. While clinical students reported less faulty oral hygiene practices, such as hard toothbrush use and aggressive toothbrushing, preclinical students reported a slightly higher mean HU-DBI score. Smoking behaviour was more common among male and clinical students, and it was also associated with alcohol drinking and worry about teeth colour and halitosis.

## Figures and Tables

**Table 1:** Demographic characteristics of Estonian dental students responding to HU-DBI; Spring 2020 (*n* = 124).

Variable	Outcome	Frequency (*n*)	Percentage (*%*)
Gender	Female	98	79%
	Male	26	21%
Academic Level	First Year	21	16.9%
	Second Year	26	21%
	Third Year	24	19.4%
	Fourth Year	23	18.5%
	Fifth Year	30	24.2%
Clinical Experience	Preclinical	47	37.9%
	Clinical	77	62.1%

**Table 2:** Health behaviours of Estonian dental students responding to HU-DBI; Spring 2020 (*n* = 124).

Variable	Outcome	Female (*n* = 98)	Male (*n* = 26)	*α*.	Preclinical (*n* = 47)	Clinical (*n* = 77)	*α*.	Total (*n* = 124)
Tobacco Smoking	Yes	5 (5.1%)	9 (34.6%)	**<0.001**	2 (4.3%)	12 (15.6%)	0.053	14 (11.3%)
	No	93 (94.9%)	17 (65.4%)		45 (95.7%)	65 (84.4%)		110 (88.7%)
Alcohol Drinking	Yes	12 (12.2%)	8 (30.8%)	**0.034** *	4 (8.5%)	16 (20.8%)	0.072	20 (16.1%)
	No	86 (87.8%)	18 (69.2%)		43 (91.5%)	61 (79.2%)		104 (83.9%)
Problematic Internet Use	Yes	85 (86.7%)	22 (84.6%)	0.754 *	36 (76.6%)	71 (92.2%)	**0.014**	107 (86.3%)
	No	13 (13.3%)	4 (15.4%)		11 (23.4%)	6 (7.8%)		17 (13.7%)
Regular Check-up	Yes	89 (90.8%)	24 (92.3%)	1.000 *	45 (95.7%)	68 (88.3%)	0.204 *	113 (91.1%)
	No	9 (9.2%)	2 (7.7%)		2 (4.3%)	9 (11.7%)		11 (8.9%)

Chi-squared test (χ2) and Fisher’s exact test (*) had been used with a significance level (α.) ≤ 0.05. All significant associations are in **bold** font.

**Table 3:** Estonian dental students’ responses to HU-DBI items; stratified by academic year; Spring 2020 (*n* = 124).

Item	Outcome	1st Year (*n* = 21)	2nd Year (*n* = 26)	3rd Year (*n* = 24)	4th Year (*n* = 23)	5th Year (*n* = 30)	*α*.	Total (*n* = 124)
No. 1	Agree	10 (47.6%)	24 (92.3%)	11 (45.8%)	20 (87%)	20 (66.7%)	0.087	85 (68.5%)
No. 2	Disagree	20 (95.2%)	25 (96.2%)	23 (95.8%)	23 (100%)	30 (100%)	0.114	121 (97.6%)
No. 3	Agree	12 (57.1%)	12 (46.2%)	17 (70.8%)	12 (52.2%)	11 (36.7%)	0.074	64 (51.6%)
No. 4	Agree	4 (19%)	5 (19.2%)	5 (20.8%)	6 (26.1%)	5 (16.7%)	0.413	25 (20.2%)
No. 5	Agree	3 (14.3%)	5 (19.2%)	0 (0%)	1 (4.3%)	3 (10%)	0.320	12 (9.7%)
No. 6	Disagree	16 (76.2%)	20 (76.9%)	21 (87.5%)	20 (87%)	26 (86.7%)	0.167	103 (83.1%)
No. 7	Agree	4 (19%)	5 (19.2%)	4 (16.7%)	3 (13%)	2 (6.7%)	0.088	18 (14.5%)
No. 8	Disagree	19 (90.5%)	24 (92.3%)	23 (95.8%)	21 (91.3%)	25 (83.3%)	0.233	112 (90.3%)
No. 9	Agree	19 (90.5%)	24 (92.3%)	24 (100%)	22 (95.7%)	29 (96.7%)	0.178	118 (95.2%)
No. 10	Disagree	17 (81%)	25 (96.2%)	17 (70.8%)	18 (78.3%)	26 (86.7%)	0.290	103 (83.1%)
No. 11	Agree	1 (4.8%)	6 (23.1%)	2 (8.3%)	3 (13%)	5 (16.7%)	0.097	17 (13.7%)
No. 12	Agree	17 (81%)	20 (76.9%)	20 (83.3%)	16 (69.6%)	22 (73.3%)	0.264	95 (76.6%)
No. 13	Agree	18 (85.7%)	13 (50%)	17 (70.8%)	15 (65.2%)	20 (66.7%)	0.062	83 (66.9%)
No. 14	Disagree	19 (90.5%)	14 (53.8%)	12 (50%)	14 (60.9%)	13 (43.3%)	**<0.001**	72 (58.1%)
No. 15	Disagree	20 (95.2%)	25 (96.2%)	24 (100%)	22 (95.7%)	30 (100%)	0.114	121 (97.6%)
No. 16	Agree	10 (47.6%)	14 (53.8%)	12 (50%)	10 (43.5%)	22 (73.3%)	**0.031**	68 (54.8%)
No. 17	Agree	4 (19%)	4 (15.4%)	2 (8.3%)	1 (4.3%)	1 (3.3%)	**0.032**	12 (9.7%)
No. 18	Agree	4 (19%)	2 (7.7%)	2 (8.3%)	0 (0%)	1 (3.3%)	**0.032**	9 (7.3%)
No. 19	Agree	10 (47.6%)	10 (38.5%)	10 (41.7%)	9 (39.1%)	9 (30%)	0.100	48 (38.7%)
No. 20	Agree	14 (66.7%)	15 (57.7%)	20 (83.3%)	20 (87%)	20 (66.7%)	0.500	89 (71.8%)

Independent-samples proportions test (one-sided) for first- vs. fifth-year students had been used with a significance level (*α*.) ≤ 0.05. All significant associations are in **bold** font.

**Table 4:** Estonian dental students’ responses to HU-DBI items; stratified by gender, clinical experience, and tobacco smoking; Spring 2020 (*n* = 124).

Item	Outcome	Female (*n* = 98)	Male (*n* = 26)	*α*.	Preclinical (*n* = 47)	Clinical (*n* = 77)	*α*.	Smoker (*n* = 14)	Nonsmoker (*n* = 110)	*α*.
No. 1	Agree	68 (69.4%)	17 (65.4%)	0.696	34 (72.3%)	51 (66.2%)	0.477	8 (57.1%)	77 (70%)	0.366 *
No. 2	Disagree	96 (98%)	25 (96.2%)	0.510 *	45 (95.7%)	76 (98.7%)	0.557 *	13 (92.9%)	108 (98.2%)	0.304 *
No. 3	Agree	50 (51%)	14 (53.8%)	0.798	24 (51.1%)	40 (51.9%)	0.924	12 (85.7%)	52 (47.3%)	**0.007**
No. 4	Agree	19 (19.4%)	6 (23.1%)	0.677	9 (19.1%)	16 (20.8%)	0.826	3 (21.4%)	22 (20%)	1.000 *
No. 5	Agree	11 (11.2%)	1 (3.8%)	0.457 *	8 (17%)	4 (5.2%)	0.056 *	1 (7.1%)	11 (10%)	1.000 *
No. 6	Disagree	81 (82.7%)	22 (84.6%)	1.000 *	36 (76.6%)	67 (87%)	0.133	13 (92.9%)	90 (81.8%)	0.461 *
No. 7	Agree	13 (13.3%)	5 (19.2%)	0.531 *	9 (19.1%)	9 (11.7%)	0.253	2 (14.3%)	16 (14.5%)	1.000 *
No. 8	Disagree	89 (90.8%)	23 (88.5%)	0.714 *	43 (91.5%)	69 (89.6%)	1.000 *	12 (85.7%)	100 (90.9%)	0.625
No. 9	Agree	93 (94.9%)	25 (96.2%)	1.000 *	43 (91.5%)	75 (97.4%)	0.199 *	12 (85.7%)	106 (96.4%)	0.137 *
No. 10	Disagree	81 (82.7%)	22 (84.6%)	1.000 *	42 (89.4%)	61 (79.2%)	0.144	14 (100%)	89 (80.9%)	0.124 *
No. 11	Agree	16 (16.3%)	1 (3.8%)	0.120 *	7 (14.9%)	10 (13%)	0.765	2 (14.3%)	15 (13.6%)	1.000 *
No. 12	Agree	78 (79.6%)	17 (65.4%)	0.128	37 (78.7%)	58 (75.3%)	0.664	12 (85.7%)	83 (75.5%)	0.517 *
No. 13	Agree	63 (64.3%)	20 (76.9%)	0.223	31 (66%)	52 (67.5%)	0.856	13 (92.9%)	70 (63.6%)	**0.034** *
No. 14	Disagree	58 (59.2%)	14 (53.8%)	0.624	33 (70.2%)	39 (50.6%)	**0.032**	7 (50%)	65 (59.1%)	0.516
No. 15	Disagree	96 (98%)	25 (96.2%)	0.510 *	45 (95.7%)	76 (98.7%)	0.557 *	14 (100%)	107 (97.3%)	1.000 *
No. 16	Agree	56 (57.1%)	12 (46.2%)	0.317	24 (51.1%)	44 (57.1%)	0.509	7 (50%)	61 (55.5%)	0.699
No. 17	Agree	9 (9.2%)	3 (11.5%)	0.714 *	8 (17%)	4 (5.2%)	0.056 *	3 (21.4%)	9 (8.2%)	0.136 *
No. 18	Agree	6 (6.1%)	3 (11.5%)	0.395 *	6 (12.8%)	3 (3.9%)	0.081 *	1 (7.1%)	8 (7.3%)	1.000 *
No. 19	Agree	37 (37.8%)	11 (42.3%)	0.672	20 (42.6%)	28 (36.4%)	0.492	6 (42.9%)	42 (38.2%)	0.735
No. 20	Agree	69 (70.4%)	20 (76.9%)	0.512	29 (61.7%)	60 (77.9%)	**0.052**	10 (71.4%)	79 (71.8%)	1.000*

Chi-squared test (*χ^2^*) and Fisher’s exact test (*) had been used with a significance level (*α*.) ≤ 0.05. All significant associations are in **bold** font.

**Table 5:** HU-DBI score of Estonian dental students and their sociodemographic and behavioural predictors; Spring 2020 (*n* = 124).

Variable	Outcome	Knowledge (0–5)	α.	Attitudes (0–3)	α.	Behaviours (0–4)	α.	HU-DBI Score (0–12)	α.
Gender	Female	4.07 ± 0.68	0.485	1.58 ± 0.72	0.146	2.51 ± 0.78	0.114	8.16 ± 1.23	0.093
	Male	4.08 ± 0.56		1.42 ± 0.50		2.31 ± 0.68		7.81 ± 1.13	
Academic Level	1st Year	4.10 ± 0.89	0.245	1.71 ± 0.64	0.376	2.38 ± 0.81	0.402	8.19 ± 1.47	0.686
	2nd Year	4.19 ± 0.63		1.54 ± 0.91		2.42 ± 0.81		8.15 ± 1.29	
	3rd Year	4.04 ± 0.46		1.46 ± 0.59		2.54 ± 0.88		8.04 ± 1.30	
	4th Year	4.04 ± 0.64		1.61 ± 0.58		2.35 ± 0.83		8.00 ± 1.09	
	5th Year	4.00 ± 0.64		1.47 ± 0.63		2.60 ± 0.50		8.07 ± 1.05	
Clinical Experience	Preclinical	4.15 ± 0.75	0.170	1.62 ± 0.80	0.207	2.40 ± 0.80	0.235	8.17 ± 1.36	0.281
	Clinical	4.03 ± 0.58		1.51 ± 0.60		2.51 ± 0.74		8.04 ± 1.13	
Tobacco Smoking	Yes	4.21 ± 0.58	0.195	1.57 ± 0.65	0.447	2.43 ± 0.76	0.419	8.21 ± 1.19	0.342
	No	4.05 ± 0.66		1.55 ± 0.69		2.47 ± 0.76		8.07 ± 1.22	
Alcohol Drinking	Yes	4.05 ± 0.61	0.433	1.50 ± 0.61	0.365	2.45 ± 0.76	0.455	8.00 ± 1.08	0.362
	No	4.08 ± 0.66		1.56 ± 0.69		2.47 ± 0.76		8.11 ± 1.25	
Problematic Internet Use	Yes	4.09 ± 0.62	0.187	1.57 ± 0.66	0.187	2.53 ± 0.74	0.008	8.20 ± 1.19	**0.006**
	No	3.94 ± 0.83		1.41 ± 0.80		2.06 ± 0.75		7.41 ± 1.23	
Regular Check-up	Yes	4.07 ± 0.66	0.923	1.54 ± 0.67	0.654	2.49 ± 0.77	0.374	8.10 ± 1.20	0.801
	No	4.09 ± 0.54		1.64 ± 0.81		2.27 ± 0.65		8.00 ± 1.41	

Two-sample *t* test (one-side) and Jonckheere-Terpstra test (*JT*) had been used with a significance level (*α*.) ≤ 0.05. All significant associations are in **bold** font.

**Table 6:** Year-over-year (YOY) analysis of HU-DBI score among Estonian dental students; Spring 2020 (*n* = 124).

Pair	Knowledge (0–5)	*α*.	Attitudes (0–3)	*α*.	Behaviours (0–4)	*α*.	HU-DBI Score (0–12)	*α*.
1st Year vs. 2nd Year	23.67/24.27	0.870	25.79/22.56	0.384	23.79/24.17	0.918	24.79/23.37	0.718
2nd Year vs. 3rd Year	27.17/23.69	0.304	26.21/24.73	0.695	24.52/26.56	0.597	26.19/24.75	0.717
3rd Year vs. 4th Year	23.94/24.07	0.968	22.35/25.72	0.341	25.56/22.37	0.396	23.52/24.50	0.798
4th Year vs. 5th Year	27.52/26.60	0.805	28.26/26.03	0.556	23.87/29.40	0.152	26.50/27.38	0.829

Mann–Whitney test (*U*) had been used with a significance level (*α*.) ≤ 0.05.

**Table 7:** Year-over-year (YOY) analysis of HU-DBI score among Estonian dental students; Spring 2020 (*n* = 124).

Predictor	Beta	S.E.	Wald	df	AOR	95% CI	*α*.
**Gender**: Male	2.18	0.71	9.35	1	8.84	2.19–35.70	**0.002**
**Experience**: Clinical Students	1.00	0.88	1.30	1	2.73	0.49–15.30	0.253
**Alcohol Drinking**: Yes	1.20	0.79	2.30	1	3.32	0.70–15.67	0.129
**Problematic Internet Use**: Yes	0.46	1.20	0.15	1	1.58	0.15–16.45	0.704
**Item No. 3**: Agree	1.69	0.90	3.53	1	5.41	0.93–31.46	0.060
**Item No. 13**: Agree	1.15	1.15	1.00	1	3.15	0.33–30.00	0.318

Logistic regression had been used with a significance level (*α*.) ≤ 0.05. All significant associations are in **bold** font.

**Table 8:** Observed and predicted group membership of tobacco smoking among Estonian dental students; Spring 2020 (*n* = 124).

Observed Group		Predicted Group		Correct Percentage
		Nonsmoker	Smoker	
Tobacco Smoking	Nonsmoker	106	4	96.4%
	Smoker	8	6	42.9%
Overall				90.3%

The cut–off value is 0.50.

## Data Availability

The data that support the findings of this study are available from the corresponding author upon reasonable request.

## Supplementary Materials

The following supporting information can be downloaded at: https://www.mdpi.com/article/10.3390/ijerph19031908/s1, Table S1: STROBE Statement—Checklist of items that should be included in reports of cross-sectional studies.

## Appendix A

**Table A1:** Modified version of Hiroshima University—Dental Behavioural Inventory (HU-DBI).

**#**	**Item**	**Agree**	**Disagree**
1	I do not worry much about visiting the dentist.	□	□
2	**My gum tends to bleed when I brush my teeth.**	□	□
3	I worry about the color of my teeth.	□	□
4	**I have noticed some white sticky deposits on my teeth.**	□	□
5	I use a child sized toothbrush.	□	□
6	**I think that I cannot help having false teeth when I am old.**	□	□
7	I am bothered by the color of my gum.	□	□
8	**I think my teeth are getting worse despite my daily brushing.**	□	□
9	**I brush each of my teeth carefully.**	□	□
10	**I have never been taught professionally how to brush.**	□	□
11	**I think I can clean my teeth well without using toothpaste.**	□	□
12	**I often check my teeth in a mirror after brushing.**	□	□
13	I worry about having bad breath.	□	□
14	**It is impossible to prevent gum disease with tooth brushing alone.**	□	□
15	**I put off going to dentist until I have a toothache.**	□	□
16	**I have used a dye to see how clean my teeth are.**	□	□
17	I use a toothbrush which has hard bristles.	□	□
18	I do not feel I have brushed well unless I brush with hard strokes.	□	□
19	**I feel I sometimes take too much time to brush my teeth.**	□	□
20	I have had my dentist tell me that I brush very well.	□	□
21	I find myself using my smartphone/computer longer than I planned.	□	□
22	I consume tobacco at least once a week.	□	□
23	I drink alcohol at least once a week.	□	□
24	I go to the dentist/hygienist for regular check-up at least once a year.	□	□

The items no. 1–20 are the original HU-DBI items, and the items in **bold** font are used to compute the overall score.
